# Erratum, Vol. 3: No. 1

**Published:** 2006-03-15

In the article "The Nutrition and Physical Activity Program to Prevent Obesity and Other Chronic Diseases: Monitoring Progress in Funded States," the following sentences had errors:

"According to self-reported weights and heights, all states had overweight rates of less than 20% for adults in 1991 (1). In 2003, the Behavioral Risk Factor Surveillance System revealed that 31 states had adult overweight rates of 20% to 24%, and four states had rates of greater than 25% (2)."

The corrected sentences are as follows:

"According to self-reported weights and heights, all states had obesity rates of less than 20% for adults in 1991 (1). In 2003, the Behavioral Risk Factor Surveillance System revealed that 31 states had adult obesity rates of 20% to 24%, and four states had obesity rates of 25% or greater (1)."

The word "overweight" was replaced with "obesity." "Greater than 25%" was replaced with "25% or greater." The second reference (2) was deleted and replaced with reference 1 (1), and the remaining references were renumbered.

The errors were corrected on our Web site on December 29, 2005, and appear online at http://www.cdc.gov/pcd/issues/2006/jan/05_0077.htm. We regret any confusion the errors may have caused.

